# Transcranial Magnetic Stimulation in Bipolar Depression: retrospective analysis and literature review

**DOI:** 10.1192/j.eurpsy.2025.2122

**Published:** 2025-08-26

**Authors:** C. P. Desport, R. P. Andrade, C. Costa, C. Gomes, A. Dias, S. N. Martins, V. Covelo, P. Valente

**Affiliations:** 1 Psychiatry, Hospital de Magalhães Lemos, Unidade Local de Saúde de Santo António, Porto; 2 Psychiatry, ULS Viseu Dão-Lafões, Viseu, Portugal

## Abstract

**Introduction:**

Bipolar affective disorder (BD) affects approximately 2% of the population. It’s an incapacitating condition that significantly impairs quality of life and functional capacity; depressive episodes in BD are highly debilitating and carry major suicide risk and treatment-resistant bipolar depression has been reported in about one-quarter of patients with bipolar disorders. Non-invasive neuromodulation procedures, such as repetitive transcranial magnetic stimulation (TMS), being an approved treatment for treatment-resistant unipolar depression, can also be an option for bipolar depression.

**Objectives:**

with this work we intend to assess the efficacy and outcomes of the intermittent theta burst TMS (iTBS) protocol in patients with bipolar depression, who underwent this treatment at Hospital de Magalhães Lemos, Porto, since July 2022. We also conducted a literature review on the subject.

**Methods:**

analysis of clinical and sociodemographic characteristics of the 4 patients who underwent treatment and of the treatment outcomes using Beck’s Depression Inventory (BDI) score difference between first and last sessions and Montgomery-Asberg Depression Rating Scale (MADRS) as the secondary outcome, the last applied to only 2 of the patients. A computerized search was performed on PubMed, for articles published in the last 10 years, using the key-words “bipolar depression”, “bipolar depressive episode” and “tms”.

**Results:**

since July 2022, 4 patients with bipolar depression were submitted to iTBS treatment, 3 women and 1 man. Of these, 3 had a diagnosis of bipolar type 1 disorder and 1 of bipolar type 2. One of the women had a comorbid diagnosis of dementia and was not able to answer BDI. All 4 of these patients were referred to this treatment after failure to reach sustained symptomatic remission with at least two different treatment trials, at adequate therapeutic doses. We found positive changes in BDI in all 3 patients that completed this questionnaire and in MADRS in the 2 that answered. One of the patients had an elevated mood and an increase in energy levels following treatment but did not meet criteria for hypomanic/manic episode. No major side effects were reported.

**Conclusions:**

Our results and literature review suggest that TMS, in our study iTBS protocol, may well be an effective treatment for bipolar depression, with some studies showing even higher response rates for bipolar depression when compared with unipolar depression, suggesting that bipolar disorder is more likely a better biological target. Furthermore, the low side effect profile of TMS and the fact that it is a minimally invasive procedure, makes it even more appealing as a treatment option. Risk of psychomotor agitation and hypomania/mania must be closely monitored in these cases.

**Disclosure of Interest:**

None Declared

